# Implementation of a high-throughput whole genome sequencing approach with the goal of maximizing efficiency and cost effectiveness to improve public health

**DOI:** 10.1128/spectrum.03885-23

**Published:** 2024-03-07

**Authors:** Michelle C. Dickinson, Samantha E. Wirth, Deborah Baker, Anna M. Kidney, Kara K. Mitchell, Elizabeth J. Nazarian, Matthew Shudt, Lisa M. Thompson, Sai Laxmi Gubbala Venkata, Kimberlee A. Musser, Lisa Mingle

**Affiliations:** 1 Wadsworth Center, New York State Department of Health (NYSDOH), Division of Infectious Diseases Bacteriology Laboratory, Albany, New York, USA; 2 Wadsworth Center, New York State Department of Health (NYSDOH), Advanced Genomic Technologies Cluster, Albany, New York, USA; University of California, San Diego, La Jolla, California, USA

**Keywords:** whole genome sequencing, NextSeq, high throughput, workflow, DNA extraction, public health, Illumina, QIAcubeHT, lab efficiency, Quarter Library Prep, Wadsworth Center, PulseNet

## Abstract

This manuscript describes the development of a streamlined, cost-effective laboratory workflow to meet the demands of increased whole genome sequence (WGS) capacity while achieving mandated quality metrics. From 2020 to 2021, the Wadsworth Center Bacteriology Laboratory (WCBL) used a streamlined workflow to sequence 5,743 genomes that contributed sequence data to nine different projects. The combined use of the QIAcube HT, Illumina DNA Prep using quarter volume reactions, and the NextSeq allowed the WCBL to process all samples that required WGS while also achieving a median turn-around time of 7 days (range 4 to 10 days) and meeting minimum sequence quality requirements. Public Health Laboratories should consider implementing these methods to aid in meeting testing requirements within budgetary restrictions.

**IMPORTANCE:**

Public Health Laboratories that implement whole genome sequencing (WGS) technologies may struggle to find the balance between sample volume and cost effectiveness. We present a method that allows for sequencing of a variety of bacterial isolates in a cost-effective manner. This report provides specific strategies to implement high-volume WGS, including an innovative, low-cost solution utilizing a novel quarter volume sequencing library preparation. The methods described support the use of high-throughput DNA extraction and WGS within budgetary constraints, strengthening public health responses to outbreaks and disease surveillance.

## INTRODUCTION

The Wadsworth Center Bacteriology Laboratory (WCBL) serves as the New York State (NYS) public health reference laboratory for bacterial identification, surveillance, and research (RES). It is a contributing member of several national surveillance programs including the Centers for Disease Control (CDC) Antimicrobial Resistance Laboratory Network (AR Lab Network, designated as the Northeastern Regional Lab), CDC PulseNet (PN) (designated as Northeastern Regional Lab), U.S. Food and Drug Administration (FDA) GenomeTrakr Network (GT), and National Antimicrobial Resistance Monitoring System Retail Food Study (NARMS). Contract work established through limited private and public partnerships (PPP) supports non-government agencies in fulfilling various project goals. Additionally, the WCBL is one of 12 state public health departments that make up the Emerging Infections Program (EIP), a national network tasked with gathering more extensive data on notifiable disease and associated risk factors. The WCBL also performs testing and contributes to programs at the state level including outbreak investigations for *Legionella* (LEG), healthcare-associated infections (HAI), and routine clinical testing for *Mycobacterium tuberculosis*.

In 2012, the WCBL began exploring the use of whole genome sequencing (WGS) with an Ion Torrent sequencer (Thermo Fisher Scientific) to achieve better resolution among strains of *Salmonella enterica* serovar Enteritidis (1). Sequencing capacity expanded in late 2013 when the WCBL joined the FDA GenomeTrakr Network, a collaboration of national and international labs focused on sequencing and publicly sharing the WGS data of foodborne pathogens from food/environmental/animal sources. FDA GenomeTrakr provided the WCBL with a Miseq (Illumina) and funds to perform WGS. By 2014, the first clinical genome of *Mycobacterium tuberculosis* was sequenced, and by 2016, WGS was being performed on all clinical cases of *M. tuberculosis* complex isolates (2). In 2015, the Wadsworth Center was selected as the CDC PulseNet Northeastern Regional Lab and became one of 10 state Public Health Laboratories (PHLs) to pilot BioNumerics 7.5 (Applied Maths/bioMérieux) software to analyze *Listeria monocytogenes* WGS data for foodborne disease surveillance. In 2017, WCBL became an AR Lab Network Regional Lab, which greatly increased sample volume. In 2019, PulseNet transitioned from Pulsed-Field Gel Electrophoresis (PFGE) as the primary laboratory method for national foodborne disease surveillance to WGS, creating a significant increase in WGS workload (3–6). In addition, other bacterial isolates that were previously tested by PFGE were also transitioned to WGS, namely, *Legionella* and those considered HAI. By the end of 2019, the WCBL was sequencing isolates for five different programs, with that number increasing to nine in 2021. A total of 5,743 genomes were sequenced from 2020 to 2021.

PHLs are continually challenged to meet the demand of increased testing capacity, often in the absence of increased funding (7). To meet the demands of increased WGS sample volume, a cost-effective, streamlined laboratory-wide workflow was developed. This paper describes the steps taken to create a workflow that incorporates a high-throughput DNA extraction platform, refined library preparation methods, and a high-throughput sequencer, NextSeq (Illumina), to reduce costs and hands-on time. The backbone of this workflow is coordination of multiple units across the WCBL, which allows the batching of large numbers of bacterial isolates requiring WGS. In 2020, the Coronavirus disease 2019 (COVID-19) pandemic strained both our instrumentation resources and available testing staff, with up to 90% of WCBL staff reassigned to the COVID-19 response. The methods described in this manuscript allowed for the continuation of all WCBL WGS, with little disruption, while facing many staffing and laboratory supply shortage obstacles (8).

## MATERIALS AND METHODS

### Sample collection and organization

As the reference laboratory for NYS, samples were referred to us from local public health departments, hospitals, clinics, health departments from other states, and any other healthcare facility requesting testing services. Pure bacterial isolates were submitted weekly to the WCBL WGS unit from all units within the Bacteriology Laboratory, except for the Mycobacteriology Unit. DNA was extracted from *Mycobacterium tuberculosis* isolates separately using an in-house developed Instagene/bead beat method (2). Genomic DNA from *M. tuberculosis* samples was added to the WCBL Illumina DNA Prep plate by the Wadsworth Center Advanced Genomic Technologies Cluster (WC AGTC) for processing. Metadata, including a unique sample identifier, genus, species, Gram stain result, genome size (Mb), and project name, were entered into an Excel Workbook, which underwent a three-tiered custom sort first by Gram stain result, then by project, and finally by sample identifier. Once samples were sorted, an extraction worksheet was created.

The Wadsworth Center Institutional Review Board has reviewed this study and determined it exempt (submission is 03-037).

### Off-board chemical and heat lysis

Two custom off-board lysis protocols allowed the pre-processing of both Gram-negative and Gram-positive bacteria before combining them in a single high-throughput DNA extraction run on a QIAcube HT (Qiagen part number 9001896), an automated DNA extraction platform. Gram-negative bacteria were lysed using a solution of 180 µL buffer ATL and 20 µL Proteinase K (50 µg/µL) per sample. Both reagents are components of the QIAamp 96 DNA QIAcube HT kit (Qiagen part number 51331). Gram-positive bacteria were lysed using 180 µL of enzymatic lysis buffer (ELB) (9) and 1.5 µL lysozyme (100 mg/mL, Sigma-Aldrich part number L6876-5G) for 30 minutes before 20 µL Proteinase K (50 µg/µL) was added for the remaining 30 minutes of incubation. Each lysis solution was created in a separate 50-mL conical tube, poured into individual sterile reagent troughs, and dispensed into a 96-well S-block (Qiagen part number 19585) using an 8-channel pipette. The extraction worksheet created during the sample organization step was used as a guide to determine which lysis solution to dispense into each well. Two wells in the S-block were reserved for reagent-only extraction controls.

A 1-µL inoculating loop was used to collect bacterial growth from each culture sample and transfer into the appropriate well in the S-block. It was crucial to only gather enough bacterial growth to just fill the 1-µL loop; there should not be a dome of growth on the loop. Processing excessive amounts of bacterial cells increased the risk of clogging the QIAamp 96 filter plate during the QIAcube HT extraction run. For any bacterial samples that were hard to collect from growth media or difficult to resuspend in the lysis solution, an inoculation needle was used to collect the bacterial growth and resuspend in the lysis solution contained in the S-block.

Once all samples were in the lysis solutions in the S-block, it was covered with an adhesive foil seal and transferred to a thermomixer. The samples were heat treated at 56°C at 300 rpm for 30 minutes. Following incubation, 20 µL Proteinase K was added to the Gram-positive samples by piercing a pipet tip through the foil seal and dispensing into the appropriate wells. A new adhesive foil seal was added, and the S-block was placed back in the thermomixer for another 30 minutes at 56°C at 300 rpm.

At the end of the 1-hour heat treatment, the S-block was left covered at room temperature for 15 minutes to minimize aerosol exposure. Next, 4 µL RNase A (100 mg/mL, Qiagen part number 19101) was dispensed into every well containing lysed samples and controls using an 8-channel pipette. After a four minute incubation at room temperature, the samples were ready to be placed on the QIAcube HT.

### High-throughput DNA extraction

The QIAcubeHT was prepared for the run with appropriate reagents from the QIAamp 96 DNA QIAcube HT kit (Qiagen part number 51331) and QIAcube HT Plasticware (Qiagen part number 950067) according to the instructions in the Setup Wizard in the QIAcube HT Software. The S-block containing sample lysates was placed on the QIAcube HT deck, and DNA extraction was performed using the Qiagen protocol “Gram-Bacterialpellets QCHT.” The program was modified so that two vacuum steps were performed during the protocol after the lysate was added to the QIAamp 96 filter plate to ensure all liquid passed through. Additionally, the QIAcube HT was programed to wait for user confirmation after both the first and second vacuum steps, so that the performing technician could visually confirm that all liquid passed through the QIAamp 96 filter plate before allowing the instrument to proceed to the next step. During the final step of the “Gram- Bacterialpellets QCHT” protocol, DNA was eluted from the QIAamp 96 filter plate with 70 µL 10 mM Tris-HCl pH 8.0.

### DNA quantification

DNA was quantified using the Quant-iT HS dsDNA Assay Kit (Thermo Fisher Scientific part number Q33120) according to the manufacturer’s instructions (10). Briefly, the Quant-iT dsDNA reagent was diluted 1:200 into the Quant-iT buffer to make enough bulk mix to measure all samples, extraction controls, and the eight dsDNA standards provided with the kit. Next, 2 µL of each DNA sample and extraction control and 10 µL of each dsDNA standard were loaded into a flat-bottom plate (Corning part number 3915) and mixed with 198 µL and 180 µL of bulk mix, respectively. After incubating at room temperature for 2 minutes, fluorescence was measured using 485-nm excitation and 538-nm emission wavelengths using a Fluoroskan Microplate Fluorometer (Thermo Fisher Scientific part number 5200110)(11). Calibration curves and DNA sample concentrations were calculated with SkanIt Software (Thermo Fisher Scientific, included with Fluoroskan). All samples with DNA concentrations between 1 ng/µL and 40 ng/µL were submitted to the WC AGTC for DNA Prep (Illumina) and sequencing. Samples greater than 40 ng/µL are diluted 1:2 before sequencing library prep.

### DNA sequencing using Illumina DNA prep quarter volume library preparation method

DNA was submitted in a 96-well plate to the WC AGTC, and NGS libraries were prepared with the Illumina DNA Prep kit (formerly Nextera Flex, part number 20060059), using a modified, quarter scale protocol. This method may be done manually or using automated liquid handlers. The WC AGTC uses both the PerkinElmer Sciclone G3 NGS Workstation (part number CLS145321) and the PerkinElmer Zephyr G3 NGS Workstation (part number CLS150362) to perform automated Illumina DNA library prep, library cleanup, and library pooling. The quarter-volume library prep programs for Sciclone and Zephyr were developed in-house with vendor support, based off a full-volume Illumina DNA prep program provided by PerkinElmer. Programing instructions for these specific liquid handlers are available upon request.

First, the Tagmentation Master Mix (TMM) was made by combining 2.5 µL of Tagment Buffer plus 2.5 µL of Bead-Linked Transposomes (BLT), per sample. Five microliters of TMM was transferred to each well of a 96-well PCR plate. Then, 7.5 µL of gDNA was transferred to the plate containing the TMM and mixed by pipetting 10 times. Molecular-grade water was added to one well to serve as the library prep no template control. The plate was sealed with a foil seal and incubated at 55°C for 15 minutes. Next, 2.5 µL of Tagmentation Stop Buffer was added to the samples, followed by a 15-minute incubation at 37°C. The 96-well sample plate was placed on a magnetic stand for 3–5 minutes to allow beads to form a pellet. While still on the magnetic stand, the supernatant was removed. Then, samples (beads) were washed two times with 35 µL Tagment Wash Buffer (TWB). The final volume of TWB was not removed until ready to proceed with the next step.

The index PCR amplification reaction required 10 µL of PCR Master Mix (5 µL Enhanced PCR Mix (EPM) plus 5 µL ddH_2_O) and 2.5 µL of Illumina Index Adapters. When automated liquid handlers were used, 2.5 µL Illumina Index Adapters were manually pipetted from the Illumina Index Adapter plate to a new 96-well PCR plate using a multichannel pipette, before being placed on the liquid handler. This is done to ensure accuracy for this low-volume transfer step. The Illumina Index plate contains enough volume for four library prep plates; thus, the plate is re-sealed with a foil seal and stored for future use.

When ready to proceed, the TWB supernatant was removed, and the plate was removed from the magnet. Then, 10 µL PCR master mix was added to the samples (beads) and mixed thoroughly to resuspend the beads. Next, the 10 µL of PCR mix and beads was transferred into a new 96-well PCR plate containing 2.5 µL of Illumina Index Adapter and mixed thoroughly. The plate is sealed and placed in the thermal cycler for the PCR step.

For manual library prep, the procedure for adding the PCR master mix and Illumina Index Adapter differed slightly. After TWB supernatant was removed, 2.5 µL of Illumina Index Adapter was transferred directly from the Illumina Index Adapter plate into the bottom of the sample plate containing the beads, without mixing. Then, 10 µL PCR master mix was added to the sample plate and mixed thoroughly to combine reagents and resuspend beads.

The Illumina BLT PCR cycling program was run on an ABI SimpliAmp or ABI 9700 thermal cycler (Thermo Fisher Scientific part numbers A24811 and 4339386, respectively), according to Illumina’s thermocycling parameters, modified by changing the number of cycles from five to seven.

Once PCR was complete, the PCR plate was placed in a centrifuge for a pulse spin to collect the samples at the bottom of the plate, then placed on a magnetic stand for 3–5 minutes until the beads formed pellets, leaving a clear supernatant containing the sample. Library cleanup and size selection were performed with Sample Purification Beads (SPB) or Illumina Purification Beads using a two-step, 0.5×/0.6× bead ratio cleanup. The SPB mix used for the two steps was made by combining 16 µL SPB with 16 µL water, multiplied by the total number of samples, plus a 20% excess. Twenty-two microliters of the SPB mix was aliquoted into each well of a fresh 96-well plate (labeled “cleanup plate 1”), and 10 µL of the SBP mix was transferred to another fresh 96-well plate (labeled “cleanup plate 2”). Next, 11 µL of sample supernatant was transferred to cleanup plate 1 to achieve the 0.5× ratio for the first step of the two-step clean up. Samples and beads in cleanup plate 1 were mixed by pipetting 10 times. The plate was sealed and put on a shaker for 1 minute at 1,600 rpm and then incubated at room temperature for 5 minutes. Next, it was placed on a magnetic stand for 3–5 minutes, until the supernatant was clear. For part two of the PCR cleanup, 30 µL of the sample supernatant from cleanup plate 1 was transferred into cleanup plate 2 and mixed by pipetting 10 times. Next, cleanup plate 2 was shaken at 1,600 rpm for 1 minute, incubated at room temperature for 5 minutes, and then placed on a magnetic stand for 3–5 minutes until the supernatant was clear. At this point, the libraries were bound to the beads, so the supernatant was removed, and the beads were washed two times with 80% ethanol. After the final ethanol was removed, the beads were allowed to air dry for 5 minutes. Cleanup plate 2 was removed from the magnetic stand, and the final libraries were eluted by adding 32 µL of resuspension buffer to each well and pipetting 10 times to resuspend the sample beads. Cleanup plate 2 was sealed with foil, shaken for 1 minute at 1,600 rpm, and then incubated at room temperature for 2 minutes. The plate was placed on a magnetic stand for 3–5 minutes to pellet the beads. Then, 30 µL of the supernatant, containing the final library, was transferred to a fresh 96-well plate.

### Library QC

Library concentrations were measured using the Quant-IT dsDNA Assay Kit HS kit on an ARVO X3 Multimode Plate Reader (Perkin Elmer) to verify successful library prep. Additionally, a subset of libraries was analyzed on an Agilent 4200 TapeStation with the High Sensitivity D5000 Assay to verify the expected library size and determine an average value for the calculation of the library molar concentrations of remaining samples (Agilent Technologies part numbers 5067-5585 and G2991BA, respectively).

### Pooling

Libraries were pooled using a custom pooling worksheet. Pooling calculations are based on genome sizes, molar concentration, and desired sequencing coverage. The pool was denatured and diluted to the standard Illumina method for NextSeq 500/550. One microliter of 20 pmol PhiX was added into the final pool for run Quality Control (QC) purposes, which results in roughly 1% PhiX reads aligned. The typical loading concentration was between 1/1 and 1.3 pmol to obtain an optimal cluster density between 170 K/mm^2^ and 220 K/mm^2^. WGS was performed on the Illumina NextSeq500 or NextSeq550 (Illumina part number SY0415-1002) sequencer using a NextSeq 500/550 Mid Output Kit v2.5 for 300 Cycles (part number 20024905). A DNA (genome) load of ~499 Mb was determined to be a reasonable amount to ensure sufficient (>40×), but not an overly excessive, average read depth for all samples on the run. For example, 80 *Escherichia coli* samples, each having a 5-Mb genome, would equal a 400-Mb total genomic load. Assuming 130M PE reads for the run, the average read depth among samples would be expected to range between 50× and 150×. Additionally, care was taken to avoid under-loading the cartridge since this could lead to excess sequencing coverage per sample. If WCBL did not submit enough samples to utilize the NextSeq cartridge capacity effectively while targeting appropriate coverage for each sample, additional samples/libraries from other labs at WC were added to the sequencing run to increase the DNA load.

### Post WGS QC and data sharing

Upon completion of the sequencing run, WC AGTC staff verify that the quality of the sequencing run meets basic QC standards (Q30 ≥ 75%, clusters passing filter ≥ 75%, and cluster density 140–240 K/mm^2^). A QC report is generated including a coverage calculation and percentage of reads passing filter for each sample, as well as the total reads passing filter for the whole run. Before bioinformatic analyses could be performed, the eight bcl files per sample were demultiplexed and converted into one R1.fastq.gz and one R2.fastq.gz file using Illumina’s bcl2fastq2 Conversion Software (hosted on a local instance of Galaxy). Converted .fastq files were parsed out according to the WCBL project code and saved on local, shared servers. Once QC analysis and bcl2fasq conversion were complete, the WC AGTC shared the QC report and filepath locations of the converted .fastq files. Each unit within WCBL performs further project-specific analyses, including, but not limited to additional QC according to parameters established by project partners (Table 1). Project-specific bioinformatic analysis, data sharing with collaborators and/or surveillance networks as appropriate, and the creation of various reports are completed by each unit as required.

**TABLE 1:** Bioinformatic analysis and QC metrics used for sequence data from each WCBL project

WCBL project	Bioinformatic platform used	QC metrics used					
		Coverage	Average quality (*Q* score)	Assembly sequence length	Contigs	Scaffolds	% core genome
ARLN	In-house-developed pipeline	Yes^*c*^	Yes^*d*^	Yes^*e*^	No	Yes^*g*^	No
GT	GalaxyTrakr, MicroRunQC	Yes^*a*^^,*e*^	Yes^*d*^	Yes^*a*^^,*e*^	Yes^*a*^^,*e*^	No	No
EIP	BioNumerics7.6	Yes^*b*^^,*e*^	Yes^*d*^	Yes^*b*^^,*e*^	Yes^*b*^^,*e*^	No	Yes^*b*^^,*e*^
HAI	In-house-developed pipeline	Yes^*c*^	Yes^*d*^	Yes^*e*^	No	Yes^*g*^	No
RES	In-house-developed pipeline	Yes^*c*^	Yes^*d*^	Yes^*e*^	No	Yes^*g*^	No
PPP	In-house-developed pipeline	Yes^*c*^	Yes^*d*^	Yes^*e*^	No	Yes^*g*^	No
PN	BioNumerics7.6	Yes^*b*^^,*e*^	Yes^*d*^	Yes^*b*^^,*e*^	Yes^*b*^^,*e*^	No	Yes^*b*^^,*e*^
RFS	BioNumerics7.6	Yes^*b*^^,*e*^	Yes^*d*^	Yes^*b*^^,*e*^	Yes^*b*^^,*e*^	No	Yes^*b*^^,*e*^
LEG	In-house-developed pipeline	Yes^*c*^	Yes^*d*^	Yes^*b*^^,*e*^	Yes^*f*^	No	No

^*a*^ (12).

^*b*^ PNQ07 and PNQ08 https://www.aphl.org/programs/food_safety/Pages/PulseNet-International SOPs.aspx.

^*c*^ All data must have ≥40X× coverage.

^*d*^ All projects require average *Q* score ≥ 30.

^*e*^ Organism dependent.

^*f*^ (13).

^*g*^ All assemblies must produce ≤500 scaffolds. RFS, Retail Food Study; ARLN, AR Lab Network.

### Cost comparison

We evaluated the total cost of WGS per sample including DNA extraction, library preparation, and sequencing. Extraction cost per sample was determined as the sum of all supplies required to perform sample extraction and DNA quantitation, including hands-on technician time, and instrument service contract cost per sample, divided by the number of samples in a run. The instrument service cost per sample was calculated by taking the average number of samples sequenced from 2020 to 2021 (2861.50), divided by the cost of an annual service contract (2021 pricing). Instrument costs were not included in this calculation. We chose to assess sample run numbers of 12, 24, 36, and 96 to determine a variety of price points based on the partial and maximum sample extraction capacity of each platform. The hands-on time and overall run time of the QIAcube Classic (Qiagen part number 9001886), QIAcube HT (Qiagen part number 9001899), and MagNAPure 24 (Roche part number 07290519001) were also recorded. Additionally, a manual extraction price analysis using the DNeasy Blood & Tissue Kit (250) (Qiagen part number 69506) was calculated when extracting 12, 24, 36, and 96 samples. This cost was calculated based on the number of samples on an extraction run multiplied by extraction cost per sample. The technician time of manual extraction was calculated by estimating the amount of time of each task of the DNeasy Blood & Tissue Kit protocol per sample and multiplying that time by the number of samples processed. In this analysis, technician time did not consider break schedules, fatigue, scheduling of tests in LIMS, submitting samples to sequencing core, reagent preparations, or DNA quantification. Library preparation and sequencing costs were based on standard billing charges from WC AGTC. This flat rate includes the cost of both Illumina and non-Illumina reagents, consumables, TapeStation analysis, cost of library preparation for controls, samples that need to be repeated, and a 15% administrative charge. All cost analyses were performed in US dollars (USD) and are based on 2021 pricing.

## RESULTS

### Evaluation of DNA Extraction Platforms

Qiagen QIAcube Classic, Qiagen QIAcube HT, and Roche MagNAPure 24 were used (Table 2). The QIAcube HT took the least amount of hands-on technician and instrument run time when processing 96 samples (5.25 hours), while also being the most cost effective when performing a run of 96 samples ($10.39 USD). A sample is defined as DNA extracted from an isolated colony of a bacterial culture growing on semisolid media. The QIAcube Classic cost per sample was similar; however. the maximum number of samples processed per run was lower. The MagNAPure 24 was the most expensive per sample ($20.33 USD per sample for a run of 24). Library preparation and sequencing costs were provided by WC AGTC, and the least expensive method they provided was the Illumina DNA Prep quarter volume library prep method (sequencing performed on the NextSeq with Mid Output v2.5-300 cycle), with a cost of $68.00 USD per sample (Table 3). WGS data derived from genomic DNA that was extracted on both the QIAcube Classic and the QIAcube HT did not show any significant sample quality differences when Q30 scores, cluster densities, and coverage were compared across the different platforms (data not shown). Due to the high cost of the MagNAPure 24, we did not process any samples on this platform; therefore, a quality comparison could not be performed. From this analysis, it was determined that the most cost-effective combination for our laboratory without sacrificing data quality was DNA extraction on the QIAcube HT (Qiagen), DNA Prep (Illumina) quarter volume library preparation, and sequencing on the NextSeq (Illumina), for a total cost of $78.39 USD per sample (genomic DNA from bacterial isolate).

**TABLE 2:** DNA extraction comparison (automated and manual): time and cost (currency in USD)^*e*^

Platform	List price	Min/max samples per run	Extraction method	Total run time^*a*^/sample cost/sample^*d*^			Pros	Cons
				Samples	Time (h)^*f*^	Cost ($)^*f*^		
QIAcube Classic	$35,360	2–12	Filter/ centrifuge	12	1.5	10.96	DNA is eluted into tubes. Customizable protocols.	Uses a lot of consumables. Max 12 samples per run.
				24	1.75	10.50		
				36	2	10.08		
				96	12	11.06		
QIAcube HT	$51,165	8–96	Filter/ vacuum	12	2	34.90	DNA eluted into 96-well plate. Customizable protocols. Process up to 96 samples per run.	Not 100% walkaway. Requires user feedback during run.
				24	2.5	21.61		
				36	3.25	17.21		
				**96**	**5.25**	**10.39**		
MagNA Pure 24	$79,500	1–24	Magnetic beads	12	1.5	22.59	DNA can be eluted into tubes. Customizable protocols.	Kits & consumables are expensive. Lengthy hands-on setup with many consumables.
				24	2.5	20.33		
				36	4.5	22.24		
				96	8.5	19.04		
DNeasy Blood & Tissue Kit	$760.00/250 reactions ($3.04/sample)	1–24^*b*^	Manual/ centrifuge	12	2^*c*^	36.48	Less consumables than automated instruments. For very small sample volumes (<12), it may be more efficient than using an automated platform.	No walkaway time, tech is at bench for the entire procedure. 100% hands-on, several transfer steps increase chance of error.
				24	4^*c*^	72.96		
				36	6^*c*^	109.44		
				96	**2 days^*c*^**	291.84		

^*a*^ Includes instrument run time (where applicable) and hands-on time.

^*b*^ Maximum manual extraction sample volume is 24 samples, with the limiting factor being a 24-position benchtop centrifuge.

^*c*^ **Bench time only**. Other factors to consider when performing manual extractions of a high sample volume are technician fatigue (extraction time may be slower as workday progresses), technician break schedule, scheduling of tests in LIMS, submitting samples to sequencing core, preparing reagents, labeling tubes, and DNA quantification.

^*d*^ Service contract price is included in the per sample cost. The service contract cost per sample was calculated by taking the average number of samples sequenced from 2020 to 2021 (2861.50), divided by the cost of an annual service contract (2021 pricing).

^*e*^ Note: Both sample cost and tech time are higher for batches of 96 samples because the amount of QIAcube Classic and MagNAPure 24 instruments available to run simultaneously is a limiting factor. Tech time will vary depending on the amount of QIAcube classics available to run at the same time. All prices are based on 2021 costs in the currency of US dollars.

^*f*^ Bold, indicates the most cost effective option.

**TABLE 3:** WC AGTC library preparation and sequencing cost comparison

Library preparation kit	Sequencing platform	Cost/sample (USD)^*b*^
Nextera XT	MiSeq-v2-500 cycle	192.00
Nextera XT	NextSeq-Mid Output v2.5–300 cycle	112.00
Nextera Flex (1/4 vol)^*a*^	MiSeq-v2-500 cycle	145.00
Nextera Flex (1/4 vol)^*a*^	Miseq-v3-600 cycle	126.00
Nextera Flex (1/4 vol)^*a*^	NextSeq-Mid Output v2.5–300 cycle	68.00

^*a*^ Nextera Flex quarter volume is an AGTC-developed protocol that uses a quarter of the full volume recommended by Illumina, plus additional modifications and optimizations.

^*b*^ Flat rate includes Illumina and non-Illumina reagents, consumables, TapeStation analysis, cost of library preparation for controls, samples that need to be repeated, and 15% administrative charge.

Additionally, a manual extraction price analysis using the DNeasy Blood & Tissue Kit (250) was calculated when extracting 12, 24, 36, and 96 samples. The cost per sample using this kit was $3.04, not including additional supplies not included in the kit. While comparable in bench time for smaller sample volumes (≥12), the use of the manual kit was more expensive than automated platforms based on price per sample alone. Additionally, there is a much greater risk for error when processing large sample volumes due to more sample handling when compared with automated platforms (there are several sample transfers in the manual method), as well as factoring in technician fatigue.

**Fig 1:**
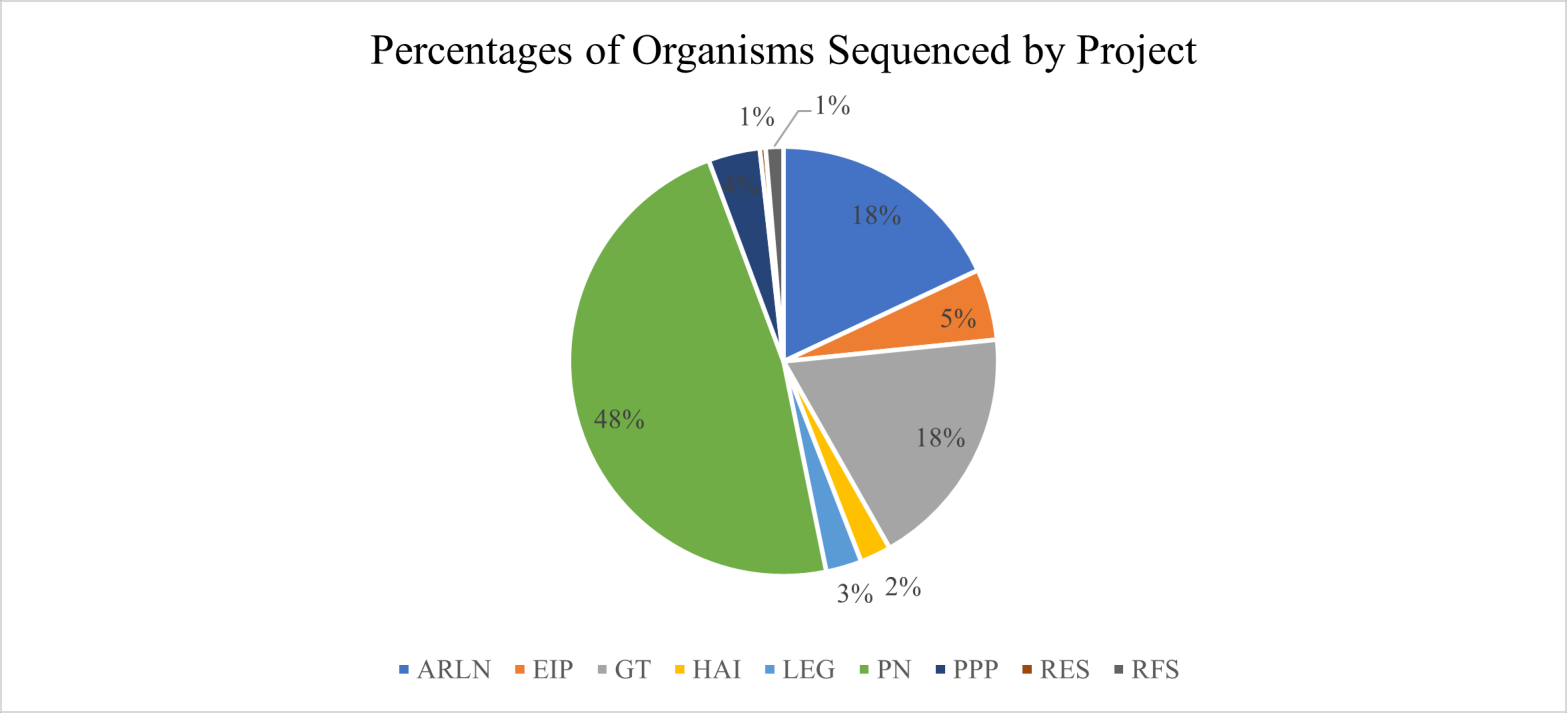
Percentages of organisms sequenced by project. AR Lab Network, Antimicrobial Resistance Laboratory Network; PPP, private and public partnerships; GT, GenomeTrakr; PN, PulseNet; EIP, Emerging Infections Program; RFS, Retail Food Study; HAI, hospital-associated infections; LEG, *Legionella* investigations; RES, Research.

From 2020 to 2021, the WCBL sequenced 5,743 genomes that contributed sequence data to nine different projects [AR Lab Network (ARLN), PPP, GT, PN, EIP, Retail Food Study (RFS), HAI, *Legionella pneumophila* investigations (LEG), and RES. Most genomes sequenced contributed to CDC PulseNet (2,741, 47%), followed by FDA GenomeTrakr (1,051, 18.3%), and AR Lab Network (1,045, 18.2%) (Fig. 1). The most sequenced organism was *Salmonella enterica* (2,336, 41%), followed by *Listeria monocytogenes* (926, 16%), *Escherichia coli* (630, 11%), *Klebsiella pneumoniae* (494, 9%), *Acinetobacter baumannii* (300, 5%), *Streptococcus pneumoniae* (246, 4%), *Legionella pneumophila* (152, 3%), *Campylobacter jejuni* (130, 2%), *Pseudomonas aeruginosa* (115, 2%), and *Enterobacter cloacae* complex (70, 1%) (Table 4). The range in genome sizes sequenced was 1.0 Mb (*Campylobacter coli*) to 8.6 Mb (*Burkholderia cepacia* complex). Twenty-three percent of the genomes sequenced was from Gram-positive organisms (1,364 genomes), while less than 1% was from anaerobic organisms (36 genomes).

**TABLE 4:** 2020–2021 sequencing workload: organisms sequenced per project^*a*^

Organism/project	Number of genomes sequenced
*Acinetobacter baumannii*	300
AR Lab Network	238
PPP	62
*Acinetobacter species*	1
AR Lab Network	1
*Aeromonas hydrophila*	2
AR Lab Network	2
*Bacillus cereus*	1
GT	1
*Burkholderia cepacia* complex	13
HAI	13
*Campylobacter coli*	16
EIP	14
PN	1
RFS	1
*Campylobacter jejuni*	130
EIP	95
PN	18
RFS	17
*Campylobacter upsaliensis*	2
EIP	2
*Citrobacter farmeri*	12
AR Lab Network	12
*Citrobacter freundii*	23
AR Lab Network	23
*Citrobacter koseri*	1
AR Lab Network	1
*Clostridium botulinum*	1
PN	1
*Clostridium perfringens*	26
GT	26
*Corynebacterium diphtheriae*	2
HAI	1
RES	1
*Corynebacterium ulcerans*	6
RES	6
*Enterobacter bugandensis*	2
AR Lab Network	2
*Enterobacter cloacae*	2
AR Lab Network	2
*Enterobacter cloacae* complex	70
AR Lab Network	66
PPP	4
*Enterococcus faecalis*	5
GT	5
*Enterococcus faecium*	1
AR Lab Network	1
*Escherichia coli*	630
AR Lab Network	96
GT	53
PN	456
PPP	25
*Haemophilus influenzae*	7
RES	7
*Klebsiella aerogenes*	3
AR Lab Network	3
*Klebsiella oxytoca*	11
AR Lab Network	11
*Klebsiella pneumoniae*	494
AR Lab Network	406
PPP	88
*Klebsiella variicola*	2
PPP	2
*Lactobacillus pentosus*	1
PPP	1
*Lactobacillus plantarum*	1
PPP	1
*Legionella pneumophila*	152
LEG	151
PPP	1
*Legionella* species	4
LEG	4
*Listeria monocytogenes*	926
GT	857
PN	69
*Neisseria gonorrhoeae*	2
RES	2
*Neisseria meningitidis*	2
RES	2
*Neisseria sicca*	3
PPP	3
*Proteus mirabilis*	5
AR Lab Network	5
*Providencia rettgeri*	6
AR Lab Network	5
PPP	1
*Pseudomonas aeruginosa*	115
AR Lab Network	81
PPP	34
*Pseudomonas oleovorans* group	**7**
AR Lab Network	7
*Pseudomonas putida* group	2
AR Lab Network	2
*Raoultella ornithinolytica*	2
AR Lab Network	2
*Raoultella* species	3
AR Lab Network	3
*Salmonella* enterica	2,336
GT	95
PN	2,180
PPP	2
RFS	59
*Serratia marcescens*	15
AR Lab Network	15
*Shigella* species	4
PN	4
*Staphylococcus argenteus*	3
RES	3
*Staphylococcus aureus*	52
AR Lab Network	4
GT	19
HAI	29
*Staphylococcus schweitzeri*	2
RES	2
*Stenotrophomonas maltophilia*	2
AR Lab Network	2
*Streptococcus pneumoniae*	246
AR Lab Network	45
EIP	197
HAI	2
RES	2
*Streptococcus pyogenes*	64
HAI	64
*Streptococcus* species	25
HAI	25
Grand total	5,743

^*a*^ AR Lab Network, Antimicrobial Resistance Laboratory Network; PPP, private and public partnerships; GT, GenomeTrakr; PN, PulseNet; EIP, Emerging Infections Program (EIP); RFS, Retail Food Study; HAI, hospital-associated Infections; LEG, *Legionella* investigations; RES, research.

Table 4 highlights how WGS data were analyzed for each bacterial species by fulfilling unique objectives for different projects. For example, 630 *E. coli* isolates were sequenced to contribute to AR Lab Network (96 genomes, 15.2%), GT (53 genomes, 8.4%), PN (456 genomes, 72.4%), and PPP (25 genomes, 4%). The WGS data were analyzed for a variety of reasons ranging from detection of novel AR mechanisms to assessing genetic relatedness for foodborne disease surveillance. Similarly, 246 *Streptococcus pneumoniae* isolates were sequenced with WGS results supporting AR Lab Network (45 genomes, 18.3%), EIP (197 genomes, 80.1%), HAI (2 genomes, 0.8%), and research (2 genomes, 0.8%).

**Fig 2:**
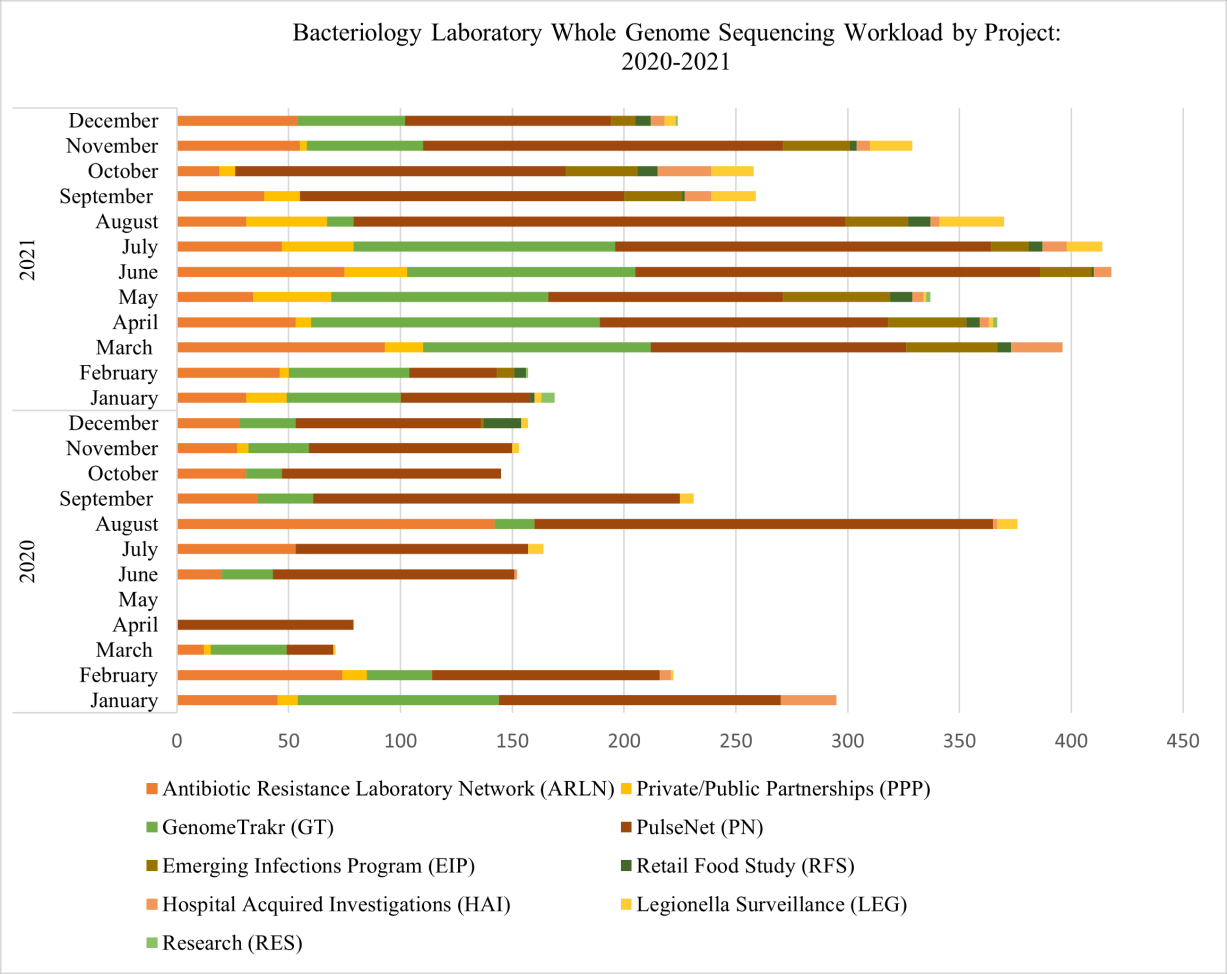
WCBL whole genome sequencing workload by project: 2020–2021.

Monthly workload showed a marked increase from 2020 to 2021 (Fig. 2). In 2020, WCBL sequenced 2,045 genomes, while 3,698 were sequenced in 2021. The 57.6% difference between samples received in 2020 versus 2021 is likely a result of the COVID-19 pandemic. During this time, the majority of WCBL staff were re-assigned to COVID-19 response efforts, research projects were suspended, and the number of samples received as part of public-private partnerships declined to nearly zero. The number of bacterial isolates sequenced for the GenomeTrakr project went from 153 samples received in January to March 2020, to just 134 samples received for the remainder of 2020. There were no genomes sequenced by WCBL in May 2020. WGS was not routinely performed during the height of the pandemic but rather performed when staff time and resources permitted. However, by March 2021, WGS sample numbers started to increase, and a greater variety of projects were being integrated into the WGS workflow. This new WGS approach allowed the WCBL to effectively handle all testing volume changes, new projects, and redirected efforts during this time period.

## DISCUSSION

PHLs are responsible for an ever-growing quantity and variety of testing capabilities. WGS is a reliable and powerful technique that can provide valuable information to enhance characterization of bacterial pathogens, including antibiotic resistance, virulence, and genetic relatedness (14). WGS has been adopted by many laboratories to generate data for local, national, and international pathogen surveillance. Here, we describe the organizational strategies, equipment, and supplies used to create a laboratory-wide flexible workflow to meet current and future needs. Coordination among different testing units in the laboratory allowed for the increase in samples needed to maximize cost effectiveness and decrease hands-on time. The most cost-effective and efficient combination of procedures for WCBL was high-throughput DNA extraction on a QIAcube HT, Illumina DNA Prep (quarter volume reactions), and sequencing on the Illumina NextSeq platform.

Coordination among different units was crucial to initiating and maintaining a collaborative, cost-effective, high-throughput system. It is important to note that, with a few exceptions, the WCBL is a reference lab that performs enhanced, confirmatory testing on reportable diseases for surveillance purposes. Therefore, it was reasonable to initiate weekly batching of pure bacterial isolates from units across the laboratory, noting that high-priority bacterial isolates could be fast tracked outside of the high-throughput workflow for sequencing on an Illumina Miseq. Sample information including sample identifier, species, Gram stain result, genome size, and project name was entered into a spreadsheet for sample tracking, library preparation, and balanced pooling of libraries.

During 2020–2021, 5,743 different bacterial genomes were processed through this single workflow, largely due to a custom off-board heat and chemical lysis procedure that enabled side-by-side processing of both Gram-negative and Gram-positive isolates in the same S-block (deep well lysis plate). Two wells on each QIAcube HT run serve as reagent-only extraction controls: one well contains 200 µL of Gram-negative lysis solution and the other contains 200 µL of Gram-positive lysis solution. These DNA extraction controls were processed through the entire QIAcube HT workflow and quantified alongside the samples at the end of the procedure. DNA extraction controls were not submitted to the WC AGTC for sequencing. Critically, we determined that a flat 1-µL loop of fresh bacterial growth was an optimal bacterial biomass that provided adequate DNA yield for Illumina DNA prep without clogging the QIAamp 96 filter plate during the QIAcube HT extraction run. We elected to program the QIAcube HT to wait for user confirmation after two critical vacuum steps to prevent sample overflow and subsequent contamination. This means that in our hands, the QIAcube HT is not a complete walk-away instrument; however, it is still a great asset owing to the benefit of performing a high-throughput DNA extraction on large numbers of diverse bacterial samples in a relatively short amount of time. The median turn-around time (TAT) from when a pure bacterial isolate was scheduled for DNA extraction to when WGS data were available for analysis in 7 days (range 4–10 days) in turn results in an approximate 2-day TAT improvement when compared with our prior workflow. This decrease is due to several reasons. (i) Low-throughput Miseq runs require more time than NextSeq runs. (ii) The high-throughput workflow takes up less technician time overall because there is one dedicated person to process all samples, rather than one person from each unit processing their units’ samples. (iii) The timetable for this workflow is very regular, excluding any unforeseen delays. Additionally, all WCBL WGS data are available for analysis in a timely and dependable manner, allowing for more consistent schedules for the WCBL staff assigned to bioinformatic analysis. This is important because the majority of WCBL staff perform multiple job roles, sometimes within different units. If there is a high sample volume on a given week, two runs can be performed so that TAT is met. Additionally, if a sample(s) needs to be rushed or is of high public health importance, WCBL has the capability to perform manual DNA extractions and sequence on a Miseq if necessary. This ability further reinforces the need for good communication between laboratory supervisors and the WC AGTC to communicate when such samples are present on a run. High-priority samples would include *Listeria monocytogenes*, meningitis cases, hospital-associated infections, shiga-toxin producing *Escherichia coli*, *Legionella pneumophila* outbreaks, and other important investigations.

The success of this workflow also depends on sequencing library preparation and the choice of sequencing instrument. The Wadsworth Center has a centralized sequencing facility with dedicated full-time staff, specialized instruments, and next-generation sequencers. Staff use automated, high-throughput liquid handlers to process samples undergoing Illumina DNA Prep. Illumina DNA Prep was chosen instead of Nextera XT for a few reasons. When using the Illumina DNA Prep, the input DNA for each sample did not have to be standardized to 2 ng/µL. Additionally, the uniformity of coverage and the quality of the sequence data were more uniform, even when using 300 cycle 2 × 150-bp sequencing chemistry (15) (). Importantly, the Illumina DNA Prep has been modified to effectively prepare sequencing libraries using quarter volume reactions. Illumina recommends 100–500 ng of input DNA to saturate the BLT and carryout full-volume reaction for Illumina DNA Prep. By scaling down to quarter volume reactions, we have reduced the required input DNA to 25–125 ng total DNA per sample. However, in our hands, DNA inputs as low as 7.5 ng have resulted in adequate library yields and produced sequence data that passes established QC thresholds. If input DNA is less than 25 ng in total and therefore the BLT is not saturated, library yields will be less and are quantified before pooling to ensure that the run is balanced and each sample gets adequate coverage.

The maximum DNA load for a NextSeq mid-output v2 reagent cartridge run for 300 cycles is 400 Mbp. For example, the NextSeq can easily sequence 80 5-Mbp genomes with greater than 40× coverage. However, the WC AGTC prepares more complex sequencing runs which contain samples with a variety of genome sizes ranging from 1 Mbp (*Campylobacter*) to >8 Mbp (*Burkholderia*), so the number of samples per run varies from week to week. WC AGTC considers the sequencing coverage requirements and genome size of all samples on the run, in addition to the DNA load. These critical adjustments resulted in $124.00 cost savings per sample, when compared with sequencing costs using the Nextera XT library preparation kit and sequencing samples on the MiSeq-v2-500 cycle platform.

The information mined from WGS data varies greatly, from bioinformatic determination of antibiotic resistance of bacterial isolates, to providing insight on healthcare-associated outbreak investigations, to assessing the genetic relatedness of bacterial isolates for ongoing foodborne outbreak investigations. As may be the case in other PHLs, the various units within the WCBL were previously siloed, owing to unique testing algorithms and downstream reporting requirements. However, now that next-generation sequencing technologies are more affordable and widely available, WGS has become a single method applied in a collaborative way to support a variety of reporting needs.

### Conclusions

PHLs should consider researching DNA extraction platforms and different next-generation sequencers based on their expected WGS sample volume. Collaboration between units within PHLs of any size can allow batching of larger numbers of samples to facilitate the use of high-throughput workflows and reduce costs. Although the workflow described in this manuscript relied on batching samples for weekly DNA extraction and sequencing, WGS data were available for analysis within 4–10 days. Future whole genome sequencing workflow improvements will focus on flexibility, such as adding fully automated NGS solutions like the Clear Labs Clear Dx system, to reduce hands on time and provide timely analysis for pathogens of public health significance.
